# Predicting in-hospital mortality in pneumonia-associated septic shock patients using a classification and regression tree: a nested cohort study

**DOI:** 10.1186/s40560-018-0335-3

**Published:** 2018-10-12

**Authors:** Jaime L Speiser, Constantine J Karvellas, Geoffery Shumilak, Wendy I Sligl, Yazdan Mirzanejad, Dave Gurka, Aseem Kumar, Anand Kumar, Institutional Review Boards (IRB) of the Cooperative Antimicrobial Therapy of Septic Shock (CATSS) Database Research Group, Yaseen Arabi, Phillip Dellinger, Sandra Dial, Peter Dodek, Paul Ellis, Daniel Feinstein, Dave Gurka, Jose Guzman, Sean Keenan, Andreas Kramer, Aseem Kumar, Stephen Lapinsky, Denny Laporta, Bruce Light, Dennis Maki, Greg Martinka, Yazdan Mirzanejad, Joseph E Parrillo, Gourang Patel, Brian Bookatz, Dan Roberts, John Ronald, Dave Simon, Yoanna Skrobik, Gordon Wood, Kenneth E Wood, Muhammed Wali Ahsan, Mozdeh Bahrainian, Rob Bohmeier, Lindsey Carter, Harris Chou, Sofia Delgra, Collins Egbujuo, Winnie Fu, Catherine Gonzales, Harleena Gulati, Oliver Gutierrez, Aparna Jindal, Erica Halmarson, Ziaul Haque, Johanne Harvey, Ehsan Koohpayehzadeh Esfahani, Farah Khan, Laura Kolesar, Laura Kravetsky, Runjun Kumar, Nasreen Merali, Sheri Muggaberg, Heidi Paulin, Cheryl Peters, Jody Richards, Honorata Serrano, Amrinder Singh, Katherine Sullivan, Robert Suppes, Leo Taiberg, Ronny Tchokonte, Omid Ahmadi Torshizi, Kym Wiebe

**Affiliations:** 1 Department of Biostatistical Sciences, Division of Public Health Sciences, Wake Forest School of Medicine, Winston-Salem, NC USA; 2 Department of Critical Care Medicine, University of Alberta, 1-40 Zeidler-Ledcor Building, Edmonton, Alberta T6G-2X8 Canada; 3 Division of Gastroenterology and Hepatology, University of Alberta, Edmonton, Canada; 4 Division of Critical Care Medicine and Infectious Diseases, University of Alberta, Edmonton, Canada; 5 Section of Critical Care Medicine, University of Manitoba, Winnipeg, Canada; 6 Section of Infectious Diseases, University of Manitoba, Winnipeg, Canada; 7 Surrey Hospital, Surrey, BC Canada; 8 Rush Medical College, Chicago, IL USA; 9 Laurentian University, Sudbury, ON Canada

**Keywords:** Pneumonia, Septic shock, Classification and regression tree, Antimicrobial therapy

## Abstract

**Background:**

Pneumonia complicated by septic shock is associated with significant morbidity and mortality. Classification and regression tree methodology is an intuitive method for predicting clinical outcomes using binary splits. We aimed to improve the prediction of in-hospital mortality in patients with pneumonia and septic shock using decision tree analysis.

**Methods:**

Classification and regression tree models were applied to all patients with pneumonia-associated septic shock in the international, multicenter Cooperative Antimicrobial Therapy of Septic Shock database between 1996 and 2015. The association between clinical factors (time to appropriate antimicrobial therapy, severity of illness) and in-hospital mortality was evaluated. Accuracy in predicting clinical outcomes, sensitivity, specificity, and area under receiver operating curve of the final model was evaluated in training (*n* = 2111) and testing datasets (*n* = 2111).

**Results:**

The study cohort contained 4222 patients, and in-hospital mortality was 51%. The mean time from onset of shock to administration of appropriate antimicrobials was significantly higher for patients who died (17.2 h) compared to those who survived (5.0 h). In the training dataset (*n* = 2111), a tree model using Acute Physiology and Chronic Health Evaluation II Score, lactate, age, and time to appropriate antimicrobial therapy yielded accuracy of 73% and area under the receiver operating curve 0.75. The testing dataset (*n* = 2111) had accuracy of 69% and area under the receiver operating curve 0.72.

**Conclusions:**

Overall mortality (51%) in patients with pneumonia complicated by septic shock is high. Increased time to administration of antimicrobial therapy, Acute Physiology and Chronic Health Evaluation II Score, serum lactate, and age were associated with increased in-hospital mortality. Classification and regression tree methodology offers a simple prognostic model with good performance in predicting in-hospital mortality.

**Electronic supplementary material:**

The online version of this article (10.1186/s40560-018-0335-3) contains supplementary material, which is available to authorized users.

## Background

Pneumonia complicated by septic shock is associated with significant morbidity and mortality. It is a leading cause of hospitalization and death with an estimated 423,000 emergency department visits per year and 15.9 deaths per 100,000 individuals in the USA [1, 2]. Annual medical costs associated with pneumonia were in excess of $10 billion annually in 2011 [3]. Most existing literature in the prognostication of pneumonia is targeted at risk stratification of patients presenting to hospital to determine the optimal location of care by predicting risk of death. Little data exists on predicting in-hospital mortality in patients presenting with pneumonia complicated by septic shock.

The primary aim of this study was to use classification and regression tree (CART) methodology to predict in-hospital mortality of patients with pneumonia complicated by septic shock. CART methodology allows the development of predictive models using binary splits on variables which can be read like a flow chart [4, 5]. Gaining popularity in diverse medical fields [6–8], CART models offer an intuitive method for predicting outcomes by using processes familiar to clinicians (e.g., “high” versus “low” values of a predictor). We hypothesized that CART models predicting in-hospital mortality would have good overall performance in terms of predictive accuracy, sensitivity, specificity, and area under the receiver operating curve (AUROC). Specifically, the objectives for this study were to:

1. Assess overall demographic and clinical characteristics of patients with pneumonia-associated septic shock
2. Compare demographic and clinical characteristics of pneumonia-associated septic shock patients based on clinical outcomes
3. Develop a CART model containing variables suggested within current literature to predict in-hospital mortality for patients with pneumonia-associated septic shock
4. Assess performance of the CART model using predictive accuracy, sensitivity, specificity, and AUROC

## Methods

This was a nested cohort study within a retrospective database (the Cooperative Antimicrobial Therapy of Septic Shock (CATSS) Database) of patients with septic shock. Data was collected from 28 medical centers in Canada, the USA, and Saudi Arabia between 1996 and 2015. The details of the study design and data collection were described in a previous paper [9]. Approval was obtained from the Institutional Review Boards of all participating institutions. This study was written according to the STROBE Guideline for reporting retrospective studies (see Additional file 1) [10].

### Study design: patients and setting

Clinical and microbiological data was extracted for all patients with pneumonia enrolled in the CATSS database. All patients in the CATSS database had septic shock, so that all patients included in our study had both pneumonia and septic shock. The diagnosis of pneumonia was made at the discretion of the physician and based on clinical, microbiological, and radiographic information. Only patients with a primary diagnosis of pneumonia were eligible for this study. Patient records and information were anonymized and de-identified prior to use in this analysis. Eligible patients with missing outcome data were excluded from the final analysis.

### Exposures and outcomes

Baseline patient characteristics including demographics and comorbid conditions were obtained at enrollment into the registry. Data collected within the first 24 h of septic shock diagnosis included serum bicarbonate level, serum lactate, bilirubin, creatinine, platelet count, international normalized ratio (INR), white blood cell (WBC) count, number of organ failures, and Acute Physiology And Chronic Health Evaluation II (APACHE II) score [11]. The primary outcome of interest was in-hospital mortality. Time to administration of appropriate antimicrobials was defined as the time of development of shock (hypotension with a mean arterial pressure < 65 mmHg and need for vasopressor support) to the time of receipt of antimicrobial therapy listed in the CATSS registry based on review of original patient records.

### Operational definitions

Septic shock was defined using the 1992 ACCP/SCCM guidelines [12]. Per that definition, patients were required to have documented or suspected infection, persistent hypotension requiring vasopressors, and at least two of the following four elements: (1) a heart rate of > 90 beats/min, (2) a respiratory rate > 20 breaths/min or arterial partial pressure of carbon dioxide (PaCO_2_) of < 32 mmHg. (3) a core temperature of < 36 °C or > 38 °C, and (4) a WBC count < 4000/μL or > 12,000/μL or bands > 10%. Hypotension was considered to represent the initial onset of septic shock when it persisted despite adequate fluid resuscitation (2 l of crystalloid) [13]. Predetermined rules were used to define documented and suspected infections and to assign significance to clinical isolates as previously described [9]. Cases of septic shock caused by infections acquired > 48 h after hospital admission were classified as nosocomial cases.

Predetermined rules were used to assess the appropriateness and delays of initial empiric antimicrobial therapy [9, 13, 14]. For septic shock with positive cultures, initial antimicrobial therapy was considered appropriate if an antimicrobial with in vitro activity appropriate for the isolated pathogen or pathogens was the first new antimicrobial agent given after the onset of recurrent or persistent hypotension or was initiated within 6 h of the administration of the first new antimicrobial agent. Initial therapy not meeting these criteria was considered inappropriate [9]. For septic shock with negative cultures, initial antimicrobial therapy was considered appropriate when an antimicrobial agent consistent with broadly accepted norms for empiric management of the typical pathogens for the clinical syndrome was the new antimicrobial agent given after the onset of recurrent or persistent hypotension or was initiated within 6 h of administration of the first new antimicrobial agent [9]. The designation of appropriateness of empiric therapy of culture-negative infections was based on recommendations listed in the “Clinical Approach to Initial Choice of Antimicrobial Therapy” from the Sanford Guide to Antimicrobial Therapy (most recently available edition at the time of the case). Additionally, infectious disease physicians and microbiologists were consulted at the discretion of the clinical team to account for local practice patterns and regional bacterial resistance patterns during the study period. To evaluate the predictive performance of the models, specificity is defined as the proportion of correctly predicted outcomes of death and sensitivity is the proportion of correctly predicted outcomes of survival.

### CART analysis

CART is a type of decision tree algorithm which follows deterministic rules to develop prediction models for continuous or categorical outcomes. This is a popular method in clinical prediction modeling because CART offers models that are simple to use with no calculations or computer applications to obtain predictions [6–8]. Additionally, CART models offer clear interpretation by using high versus low values of clinical variables related to the outcome of interest based on optimal splitting criteria from an automated algorithm. Trees are read from top to bottom like a flow chart in order to obtain a prediction for a specified outcome (e.g., survived or died). Starting at the top of a tree, branches corresponding to observed clinical features are followed until a terminal node has been reached and the fraction of patients contained in each outcome group is displayed. These tables may be used to assess the probability that a patient falls within each outcome category.

CART models were developed using the following algorithm first introduced by Breiman [4].Trees were constructed firstly by selecting the variable that optimally separated outcome groups, and a binary split was made. Then, from both of these subgroups, subsequent variables were selected with replacement (meaning that variables can be used more than once within a model) that optimally separated outcome groups, and second levels of binary splits were made. Variable splits were made recursively until stopping criteria were reached, in which case a terminal node occurred. At each terminal node was the outcome prediction for the specific subset of the data.

The features of CART described in the previous paragraph are advantageous compared to standard logistic regression for modeling binary outcomes. Potential deficiencies of logistic regression for clinical prediction modeling include cumbersome calculations (e.g., inserting numbers and exponentiation requires a calculator or application), unclear interpretation of results (e.g., log odds ratios are not intuitive, especially in the presence of interactions between predictor variables), and unsatisfied assumptions (e.g., linear relationship between predictors and outcome via the link function may be inappropriate). CART also includes a method for handling missing predictor data using surrogate splits while logistic regression requires missing data to be filled in using a separate imputation method for all observations prior to developing a prediction model. For these reasons, CART is a beneficial framework for developing clinical prediction models compared to logistic regression.

### Variables

The main outcome of interest was in-hospital mortality. Multiple variables were used in developing the prediction model. Clinical variables included age, sex, use of mechanical ventilation, location of infection acquisition (nosocomial or community), underlying immunosuppression, number of systems with end-organ dysfunction, time to appropriate antimicrobial therapy, body mass index, and APACHE II score. Biochemical variables included serum lactate, bilirubin, sodium, creatinine, INR, platelets, WBC count, and albumin. Microbiological variables included culture positivity, concomitant bacteremia/fungemia, isolated fungal and bacterial pathogens, and the presence of antimicrobial resistant organisms. All clinical predictors were collected at baseline unless otherwise noted.

### Statistical methods

Analyses were completed using RStudio software [15]. Patient characteristics were presented as mean (standard deviation (SD)) or *n* (percent) and compared using *t* tests and binomial tests using the R package *tableone* [16]. *P* values of < 0.05 were considered statistically significant. CART models were constructed using a training dataset (*n* = 2111) and were assessed using a testing dataset (*n* = 2111). Training and test data were randomly split from the entire dataset. The R package *rpart* was used to develop the CART models [17]. Missing predictor data were handled using the method of surrogate splitting, which is a standard built-in feature of CART implementation using the *rpart* package. CART can sometimes produce models, which overfit data (i.e., they can model too many splits for a specific training dataset), which may not predict well for independent test data. One of the ways to reduce overfitting is by constraining the number of observations, which each terminal node of the tree must contain. We required that the minimum number of observations in terminal nodes of the CART was 100 (i.e., the tuning parameter for minimum bucket size was 100) to provide a sufficient amount of data relative to the total training sample for meaningful predictions within the final variable splits. Prediction models were assessed in terms of overall accuracy, sensitivity, and specificity using binomial estimates and confidence intervals. AUROC and its corresponding confidence intervals were determined using the R packages *ROCR* [18] and *cvAUC* [19].

## Results

### Overall demographic and clinical characteristics

In total, 4222 patients (61% male) with pneumonia and septic shock were included in the analysis (Table 1). The mean (SD) age of patients was 62 (17) years. Sixty-three percent (*n* = 2652) had positive cultures from clinical isolates, 21% (*n* = 876) had concomitant bacteremia, and 35% (*n* = 1075) had nosocomial infections. Of patients with positive cultures, the most common pathogens were *Staphylococcus aureus* (*n* = 702, 27%), *Streptococcus* spp. (*n* = 658, 25%), *Pseudomonas* spp. (*n* = 267, 10%), *Escherichia coli* (*n* = 225, 9%), *Klebsiella* spp. (*n* = 183, 7%), and *Haemophilus influenzae* (*n* = 118, 4.4%). Mean (SD) APACHE II score was 26 (8), and serum lactate was 4.1 (3.9) mmol/L at onset of septic shock. During ICU admission, 89% (*n* = 3760) required mechanical ventilation. Of 3048 patients who received appropriate antimicrobial therapy after the development of septic shock, the mean time to administration of antimicrobials was 10.9 h (SD = 18.6 h). Fifty-one percent (*n* = 2141) of patients died in hospital.

**Table 1:** Demographic and clinical characteristics of pneumonia-associated septic shock patients

	Overall cohort (*n* = 4222)	
	*N*	Number (%) or mean (SD)
Demographics		
Age	4222	62 (17)
Sex (male)	4222	2574 (61.0)
Body mass index	2013	27 (8)
Microbiology characteristics		
Concomitant bloodstream infection	4222	876 (20.7)
Empyema	4222	119 (2.8)
Culture positive	4222	2652 (62.8)
Gram positive	4222	1413 (33.5)
Gram negative	4222	1073 (25.4)
Fungal	4222	20 (0.8)
Hospital-acquired infection	4222	1547 (36.6)
Community-acquired infection	4222	2675 (63.4)
Organ failure/support		
APACHE	3995	26 (8)
Organ failure day 1	4222	3.8 (1.5)
Mechanical ventilation	4222	3760 (89.1)
Biochemistry (admission)		
WBC	4031	16.3 (15.7)
Platelets	4046	206 (136)
Sodium	2488	137.2 (7.1)
Creatinine	3829	189.9 (164.6)
Lactate	2804	4.1 (3.9)
INR	3695	1.7 (1.3)
Bilirubin	3544	29.9 (64.6)
Albumin	1506	22.7 (6.5)
Immunocompromised	4222	561 (13.3)
Time delay from shock to appropriate antimicrobials (hours)	3048	10.9 (18.6)
Primary outcome: in-hospital mortality	4222	2141 (50.7)

Of patients with pneumonia and septic shock, 2141 died in hospital and 2081 survived. Patients who died in hospital were significantly older (mean age of 65 versus 59) and had lower body mass index (28 versus 27) when compared to survivors (Table 2). The presence of concomitant bloodstream infection, empyema, positive microbiology, gram-negative pathogens, and fungal pathogens were associated with increased in-hospital mortality. Nosocomial pneumonia infections, higher APACHE II scores, and higher numbers of organ failures were also associated with worse outcomes (Table 2). Mechanical ventilation was more commonly used in patients who died. Admission biochemistry revealed that patients who died had significantly lower platelets, higher lactate, higher INR, higher bilirubin, and lower albumin compared to patients who survived. There was no significant difference detected between the groups for white blood cell count, sodium, and creatinine. In-hospital mortality was significantly more common in patients who were immunocompromised. The mean time to administration of appropriate antimicrobial therapy was 5 h in patients who survived and 17 h in patients who died.

**Table 2:** Demographic and clinical characteristics of pneumonia-associated septic shock patients by mortality

	Died (*n* = 2141)		Survived (*n* = 2081)		*P* value
	*N*	Number (%) or mean (SD)	*N*	Number (%) or mean (SD)	
Demographics					
Age	2141	64.6 (15.8)	2081	58.8 (16.7)	< 0.001
Sex (male)	2141	1323 (61.8)	2081	1251 (60.1)	0.277
Body mass index	974	26.6 (7.8)	1039	27.7 (7.7)	0.001
Microbiology characteristics					
Concomitant bloodstream infection	2141	484 (22.6)	2081	392 (18.8)	0.003
Empyema	2141	45 (2.1)	2081	74 (3.6)	0.010
Culture positive	2141	1421 (66.4)	2081	1231 (59.2)	< 0.001
Gram positive	2141	696 (32.5)	2081	717 (34.5)	0.191
Gram negative	2141	608 (28.4)	2081	465 (22.3)	< 0.001
Fungal	2141	16 (0.7)	2081	4 (0.2)	0.009
Hospital-acquired infection	2141	957 (44.7)	2081	590 (28.4)	< 0.001
Organ failure/support					
APACHE	2034	28.5 (8.0)	1961	22.8 (6.7)	< 0.001
Organ failure day 1	2141	4.2 (1.6)	2081	3.4 (1.3)	< 0.001
Mechanical ventilation	2141	2005 (93.6)	2081	1755 (84.3)	< 0.001
Biochemistry (admission)					
WBC	2052	16.3 (17.9)	1979	16.4 (13.0)	0.757
Platelets	2021	195 (143)	2025	216 (128)	< 0.001
Sodium	1121	137.4 (7.2)	1367	137.0 (7.0)	0.192
Creatinine	1937	192.2 (164.6)	1892	187.5 (164.6)	0.377
Lactate	1447	5.1 (4.6)	1357	3.1 (2.8)	< 0.001
INR	1848	1.9 (1.5)	1847	1.6 (1.1)	< 0.001
Bilirubin	1768	39.7 (82.7)	1776	20.1 (36.7)	< 0.001
Albumin	621	21.6 (6.3)	885	23.5 (6.4)	< 0.001
Immunocompromised	2141	360 (16.8)	2081	201 (9.7)	< 0.001
Time delay from shock to appropriate antimicrobials (hours)	1494	17.2 (23.6)	1554	5.0 (5.6)	< 0.001

### CART model predicting in-hospital mortality

**Fig. 1:**
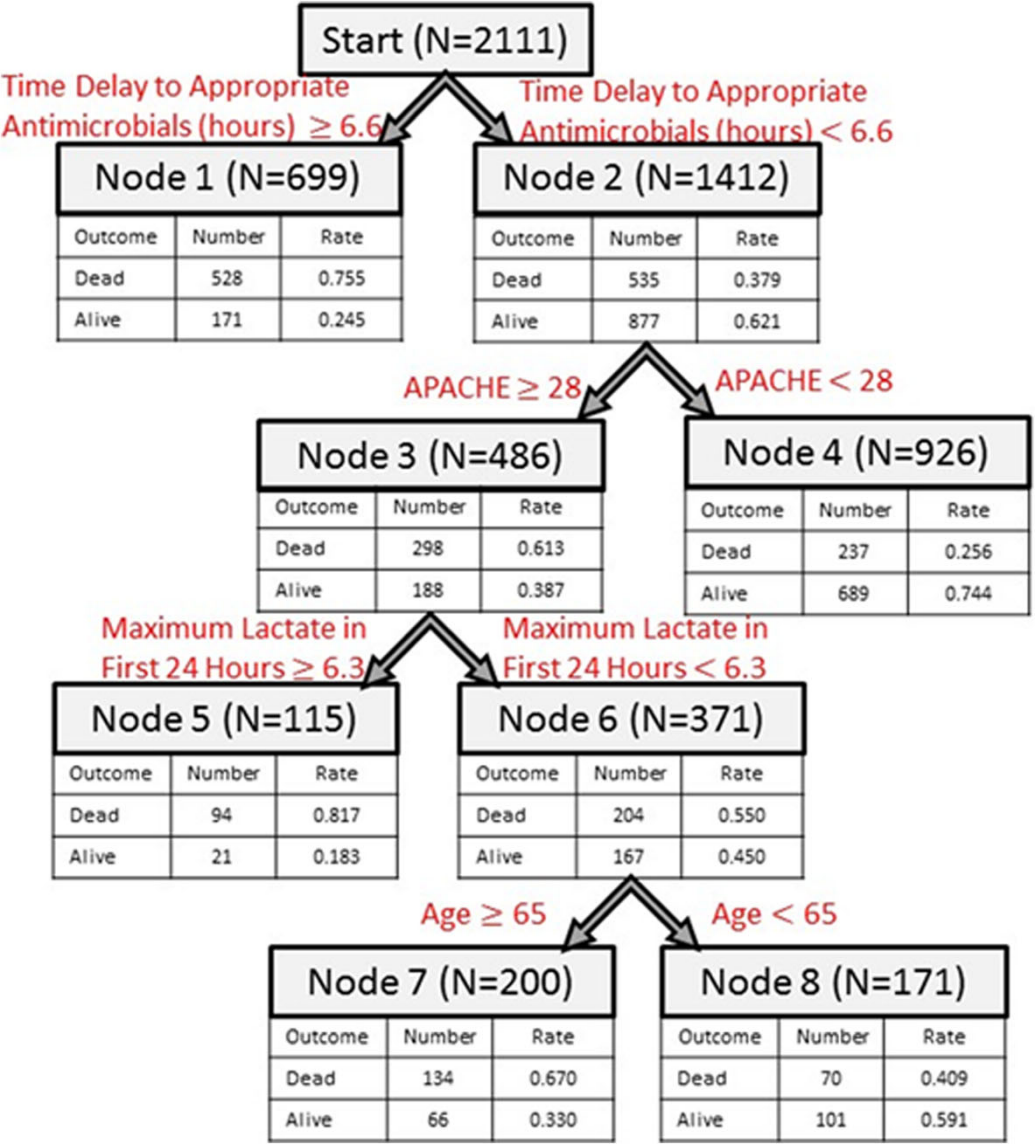
Depicts the resulting classification and regression tree for predicting in-hospital mortality. The decision tree contains four predictors: time to appropriate antimicrobial therapy, APACHE II score, lactate, and age. Terminal nodes containing predictions for new observations include 1, 5, and 7 (predict death) and 4 and 8 (predict alive). To obtain a prediction, one starts at the top of the tree and follows the arrow corresponding to data for the new observation until a terminal node is reached

The overall dataset was randomly split into training data for model development and testing data for model validation. There were no significant differences detected between the training and test datasets. The CART model for predicting mortality in patients with pneumonia and septic shock is depicted in Fig. 1. Variables included within the model were the time to administration of appropriate antimicrobial therapy, APACHE II score, serum lactate, and age. The most important predictor of in-hospital mortality was the time to appropriate antimicrobial therapy.

The following features were associated with higher probability of death:

1. Time from onset of septic shock to administration of appropriate antimicrobial therapy > 6.6 h (node 1, probability of death = 0.76)
2. Time from onset of septic shock to administration of appropriate antimicrobial therapy < 6.6 h, APACHE > 28, and lactate > 6.3 mmol/L (node 5, probability of death = 0.817)
3. Time from onset of septic shock to administration of appropriate antimicrobial therapy < 6.6 h, APACHE > 28, lactate < 6.3 mmol/L, and age > 65 (node 7, probability of death = 0.670)

The following features were associated with higher probability of survival:

1. Time from onset of septic shock to administration of appropriate antimicrobial therapy < 6.6 h and APACHE < 28 (node 4, probability of survival = 0.744)
2. Time from onset of septic shock to administration of appropriate antimicrobial therapy < 6.6 h, APACHE > 28, lactate < 6.3 mmol/L, and age < 65 (node 8, probability of survival = 0.591)

There were 1174 patients who received appropriate antimicrobials before the onset of septic shock. In the training dataset used to develop the CART prediction model, these were treated as missing. The CART framework uses a method called surrogate splitting in order to handle any missing values, in which non-missing variables are used to make a “surrogate” split for any missing values. Thus, the patients who received appropriate antimicrobials before onset of septic shock were included in the CART model development. For use in practice for new observations of patients, one should follow the branch corresponding to time to appropriate antimicrobials < 6.6 within Fig. 1 (i.e., proceed to node 2).

### Predicting in-hospital mortality: an example

A patient with pneumonia and septic shock presented at the hospital with the following characteristics: antimicrobials were administered 3 h after septic shock, APACHE II score of 30, lactate of 10.2 mmol/L, and age of 64. At the start, time to antibiotic administration is less than 6.6 h (true at node 1), so we follow the right branch to node 2. Next, the APACHE II score is > 28 is true, so we follow the left branch to node 3. Then, lactate is > 6.3 mmol/L, so we proceed to the left branch to node 5. Since there are no nodes under node 5, this is our final prediction for the model. The probability of death for the patient is 0.817, and the probability of survival is 0.183. Therefore, the patient is predicted to die in-hospital.

### Assessing performance

Performance measures and the associated confidence intervals for the CART model are presented in Table 3. In the training dataset, the CART prediction model for mortality yielded overall accuracy of 73%, specificity of 75%, and sensitivity of 71%. The model showed good overall performance, with training dataset AUROC of 0.75. In the testing dataset, the CART prediction model for mortality yielded accuracy of 69%, specificity of 72%, and sensitivity of 65%. The model had good overall performance, with testing dataset AUROC of 0.72.

**Table 3:** Performance measures (95% exact binomial confidence intervals) for the CART model prediction in-hospital mortality

Model	Accuracy (95% CI)	Specificity (95% CI)	Sensitivity (95% CI)	AUROC (95% CI)
Training (*n* = 2111)	0.73 (0.71, 0.75)	0.75 (0.73, 0.78)	0.71 (0.68, 0.74)	0.75 (0.73, 0.78)
Testing (*n* = 2111)	0.69 (0.67, 0.71)	0.72 (0.70, 0.75)	0.65 (0.62, 0.68)	0.72 (0.69, 0.75)

## Discussion

### Summary of key results

In this study, we evaluated a large multi-center cohort of patients with pneumonia complicated by septic shock. Overall mortality (51%) was high in this population. There were 3048 patients who received appropriate antimicrobial therapy after the development of septic shock with a mean time to appropriate antimicrobial therapy of 10.9 h. Patients who died in the hospital were significantly older and had significantly higher APACHE II scores, number of organ failures, and admission serum lactate. Time to administration of appropriate antimicrobial therapy remained the most important predictor of in-hospital mortality in this population. In the training set (*n* = 2111), a CART model using APACHE II score, lactate, age, and time to appropriate antimicrobial therapy yielded predictive accuracy of 73%, specificity 75%, sensitivity 71%, and AUROC 0.75. In the testing set (*n* = 2111), the CART model offered predictive accuracy of 69%, specificity 72%, sensitivity 65%, and AUROC 0.72.

The novelty of the study is the use of classification and regression tree (CART) methodology for the development of a simple, accurate prediction model for outcomes in pneumonia patients with septic shock. CART allows for development of prediction models using binary splits and offers an intuitive method for obtaining predictions of outcome using processes familiar to clinicians (e.g., “high” versus “low” values of a predictor). The nonparametric nature of CART offers results that are simple to use and does not require calculation of use of an application. Models are easily read and interpreted using a flow chart diagram. These aspects of CART are advantageous compared to logistic regression, where calculations may be cumbersome (e.g., plugging in numbers and exponentiation requires a calculator or application), interpretation of results may be unclear (e.g., if there are interactions between two or more predictors), and assumptions may not be satisfied.

### Comparison with the literature

Patients with pneumonia complicated by septic shock are at substantial risk of poor outcomes. The 51% in-hospital mortality observed in this cohort is substantially higher than the reported mortality for population-level outcomes in patients with pneumonia and patients presenting with pneumonia and septic shock [20, 21]. Despite these studies, a lack of data on predicting outcomes for patients with pneumonia and septic shock remained.

In this study, a mechanism for predicting the probability of in-hospital mortality was developed using CART methodology. Previous prediction models have focused on predicting patient outcomes for purposes of risk stratification at presentation to hospital with pneumonia [22–24]. Consistent with previous literature, our study highlights that the presence of septic shock and the severity of illness (APACHE II), age, lactate, and time to administration of appropriate antimicrobial therapy significantly impacts survival in patients with pneumonia. In our study, multivariable CART analysis demonstrated that the most important predictor of mortality was the increasing time from onset of septic shock to administration of appropriate antimicrobial therapy. Additional predictors of in-hospital mortality included severity of illness (APACHE II score), high serum lactate, and older age. Our study complements previous research by highlighting the importance of early intervention and administration of appropriate antimicrobial therapy to optimize outcomes in patients with septic shock.

Though CART models have existed for several decades, there is a paucity of decision tree models available for predicting outcomes in critically ill patient populations. Wong et al. [25] use CART to analyze 355 children with septic shock to assess biomarkers and clinical variables. The resulting decision tree consisted of five biomarker-based decision rules with ten variable splits. This work was primarily done to complement microarray work to explore potential gene products as targets in sepsis. Wong et al. subsequently applied the same five biomarkers along with lactate, age, and chronic disease status [26] in 672 adult patients with septic shock with and developed a clinical prediction model with an area under the receiver curve of 0.72 (validation set), similar to this study. Besides these two studies which primarily focused on gene products/potential novel biomarkers (both < 700 patients), the decision tree approach for prediction has not been previously used for a large population of adults with septic shock using readily available clinical information as in this study.

### Limitations

This study should be interpreted within the limitations of its design. This study is a retrospective analysis of prospectively collected data and only association, not causation, can be inferred. Given that this study was observational, we are unable to conclusively exclude sources of selection bias [27]. We implemented an internal validation scheme that used randomly split training and testing datasets to build and evaluate the CART prediction model for mortality. External data should be used to further validate the CART model. Another limitation is that the average time to appropriate antimicrobial therapy was 6 h, which is greater than the current suggested 3-h completion of treatment. These guidelines changed over the course of the study period (from 1996 to 2016), so we included all data in order to have a larger sample size to develop a prediction model. Though current guidelines suggest completion of the sepsis bundle within 3 h [28], approximately one third of the patients in this study received appropriate antibiotic after 6 h. There are several reasons for the longer time to antibiotics: our study included appropriate use of antibiotics not just time to any usage of antibiotics, about half of the patients included in our study were ward patients which have higher time to appropriate antimicrobials compared to emergency room admissions, and the data range for our study is from 1996 to 2016 during which the time to antibiotics was substantially longer than the standard practice now. Despite these limitations, the strengths include inclusion of patients from 28 intensive care units across three geographic regions.

Limitations of CART modeling include the challenge of determining parameters for model building (e.g., deciding the minimum bucket size) and the possible variability of CART models, as discussed in statistical literature (e.g., [8, 29–32]) (Additional file 2). Inclusion of variables for age and APACHE II, which also used age for its calculation, highlights the importance of age for predicting outcomes of pneumonia-associated septic shock patients. Unlike traditional regression models, which can be negatively influenced by correlated variables, the CART model can adequately handle correlated variables due to the binary nature of splitting. However, these limitations are minimal compared to the beneficial simplicity and relatively high predictive accuracy of CART models.

## Conclusion

Overall mortality in patients with pneumonia and septic shock is high (51% in the CATSS dataset). Increasing time to appropriate antimicrobial therapy, APACHE II score, serum lactate, and age were significantly associated with in-hospital mortality. CART models offer simple prognostic models with good performance.

## Additional files

Additional file 1: STROBE guideline for reporting retrospective studies. (DOCX 38 kb)

Additional file 2: Benefits of CART, tree development, and limitations of CART models. (DOCX 17 kb)

## Abbreviations

APACHEII: Acute Physiology and Chronic Health Evaluation II score
AUROC: Area under the receiver operating curve
CART: Classification and regression tree
CATSS: Cooperative Antimicrobial Therapy of Septic Shock
CI: Confidence interval
ICU: Intensive care unit
INR: International normalized ratio
SD: Standard deviation
WBC: White blood cell
