# Supplementary material for: Predicting in-hospital mortality in pneumonia-associated septic shock patients using a classification and regression tree: a nested cohort study
Source: J Intensive Care. 2018 Oct 12;6:66. doi: 10.1186/s40560-018-0335-3 (PMC6186142; doi:10.1186/s40560-018-0335-3)
Supplement: Supplementary file 2 — Benefits of CART, tree development, and limitations of CART models. (DOCX 17 kb) [file 40560_2018_335_MOESM2_ESM.docx]

**Additional file 2: Supplementary methods**

**Benefits of CART**

Constructing optimal binary splits on independent variables to predict outcome, CART is simple to apply in practice and offers reliable accuracy. Compared to traditional categorical prediction models such as logistic regression and linear discriminant analysis, CART has few statistical assumptions. No requirements about the distribution of variables are necessary within this framework. CART also provides an effective way of handling observations with missing predictors in the data and allows for inclusion of variables with complex interactions and correlations. Modern advances in computing make CART modeling efficiently implemented using many standard statistical software platforms.

**Tree Development**

Trees were constructed firstly by selecting the variable that optimally separated outcome groups, and a binary split was made. Then, from both of these subgroups, subsequent variables were selected with replacement (meaning that variables can be used more than once within a model) that optimally separated outcome groups, and second levels of binary splits were made. Variable splits were made recursively until stopping criteria were reached, in which case a terminal node occurred. At each terminal node was the outcome prediction for the specific subset of the data.

**Limitations of CART Models**

Though CART models offered an alternative to current prediction models (e.g. regression), there are some limitations of this method. Firstly, CART models can create models, which are complex, and users must decide how to prune models, which may introduce bias. Using cross-validation, our CART model performed only slightly worse for the test dataset compared to the training dataset, which indicated that bias is likely minimal. Also, CART may have more variability compared to more complex statistical algorithms, such as ensemble models. This creates a more consistent prediction method, although the price for model stability is diminishing simplicity and interpretability. CART dominates ensemble methods in terms of simplicity, while still providing impressive accuracy and predictive performance.
